# Cases of Dislocation of the Clavicle Backwards

**Published:** 1842-04

**Authors:** 


*Cases of Dislocation of the Clavicle backwards behind the Sternum.* By M. Morel.—Case I. Lemoine, 17 years of age, was overtaken in a narrow street by a carriage, and had his right shoulder violently squeezed against the wall, in a direction forwards and inwards. He experienced at the time some pain at the bottom of the neck, and a great sensation of suffocation which lasted more than a quarter of an hour. The dyspnœa gradually subsided, but the motion of the right arm not returning, he, on the 8th day after the accident, entered the *Charité*, when he was found in the following state:—The two shoulders were on the same level, but the right one was nearer the mesial line. The internal extremity of the clavicle was half concealed behind the sternum, and its upper part consequently formed a projection above the bone. When the shoulder was carried backwards the tumor became more prominent forwards. On depressing the shoulder, it rose, and disengaged itself from behind the sternum, and, by carrying the shoulder upwards, outwards, and backwards, the bone returned to its natural position; the hand could be carried to the head, by directing the elbow backwards, but if it was brought forwards, the attempt caused great pain. Desault's bandage was applied; but, as it slipped, it was replaced by that of Velpeau, which kept the bone completely in position. The patient was lost sight of on the 18th day, as he was obliged to be removed to another ward on account of the appearance of smallpox.

Case II.—Lamotte, 28 years of age, was shoeing a horse, when the animal made violent efforts to free his leg; endeavoring to restrain them, he fell, and met with a dislocation of his clavicle backwards. The next day he was in the following state:—The right shoulder was nearer by sixteen lines, than usual, to the mesial line; there was slight swelling of the external jugular vein; some pain was felt, but more towards the middle than the extremity of the clavicle, the external head of which had disappeared behind the sternum, where it was impossible to feel it. When the hand was carried to the head, there was pain if the elbow was moved forwards, but none if it was directed backwards. Two attempts at reduction made, the one by carrying the shoulder outwards and backwards, the other by acting on the arm in the same direction, were unsuccessful.

M. Lenoir made the patient be seated on a low chair; he then placed a sheet round his body, and fastened it to an iron bar. Two assistants pulled the arm and shoulder outwards and backwards, (almost horizontally in fact,) while another kept the wrist lowered, and at the same time turned towards the left side. M. Lenoir then placed his knee between the shoulders, and with one hand pulled the right one backwards, while with the other he traced the clavicle. Reduction was accomplished, and the bone was retained in its place by the shoulders being kept back by a bandage applied in the form of the figure 8. Another bandage was placed round the body, and kept the elbow close to the side. On the twelfth day this was removed and a sling had recourse to. On the eighteenth day he was doing well. The reduction was so complete, that the extremity of the dislocated clavicle was more prominent than that of the other.

The first case put on record of this kind of dislocation, caused by violence, was published by M. Pellieux in 1834, in the Revue Médicale. Since then two other cases have been reported in the Gazette Médicale for 1836–37. Besides the two that we have given above, a third is quoted by the Gazette Médicale, from M. Morel's pamphlet; but we have not given it, as it appears to us not to be a case of dislocation backwards. In Sir A. Cooper's Lectures on Surgery, a case is mentioned, which was produced by deformity of the spine. The scapula was pushed forward, so that sufficient room was not left for the clavicle between that bone and the sternum, and its internal head gradually slipped behind the latter, pressing upon the œsophagus, and giving rise to great difficulty of swallowing. In this case, as reduction was impossible, the clavicle was sawn through, about an inch from the sternum, and dissected out. The operation was done by Mr. Davis, late of Bungay, in Suffolk. From the cases published, M. Morel says, that there are two kinds of this dislocation, viz. backwards and downwards, and backwards and upwards, as when a part of the inner head of the clavicle is above the level of the sternum. We can suppose that the latter species is consecutive, and produced by the action of the sterno-mastoid muscle, and by the weight of the superior extremity. The dislocation downwards is much more difficult than the other to reduce, and when reduction is effected, the bones are not so easily retained in their place.—*L. and E. Monthly Jour. Med. Sci.*, from *Gazette Médicale,* 21st Aug. 1841.

